# Precision Control in Vat Photopolymerization Based on Pure Copper Paste: Process Parameters and Optimization Strategies

**DOI:** 10.3390/ma16165565

**Published:** 2023-08-10

**Authors:** Weiqu Wang, Mengzhao Feng, Zhiwei Wang, Yanlin Jiang, Bohang Xing, Zhe Zhao

**Affiliations:** 1 School of Materials Science and Engineering, Shanghai Institute of Technology, Shanghai 201418, China; wangweiqu217@163.com (W.W.); fmz206081112@163.com (M.F.); ggwangboy@163.com (Z.W.); 2 Jiaxing CeramPlus Technology Co., Ltd., Jiaxing 314100, China; jyanlin2023@163.com

**Keywords:** additive manufacturing, vat photopolymerization, copper paste, dimensional accuracy, ANOVA

## Abstract

Vat photopolymerization (VPP) presents new opportunities for metals to achieve the design freedom of components. However, the material properties of copper powder and the inherent defects of the technology seriously hinder its application in high-precision metal additive manufacturing. Precision control is the key to obtaining minimal precision metal parts when copper is prepared by reduction photopolymerization. This paper employed variance analysis (ANOVA) and root mean square deviation (RMSD) to determine the significant parameters affecting dimensional accuracy and their optimal regions. The results show that printing accuracy is improved by optimizing exposure time, intensity, layer thickness, and sweeper moving speed. When the exposure time is 21 s, and the exposure intensity is 220 mW/cm^2^, a hole with a height of 1 mm and a diameter of 200 μm can be printed with a minimum size deviation of 51 μm. In addition, RMSD and ANOVA provide an effective method for realizing high-precision stereolithography 3D printing metal copper, expanding the material adaptation in the 3D printing metals field. The study highlights the potential of VPP as a method for preparing metals in future studies.

## 1. Introduction

Structural metals based on pure copper exhibit excellent electrical and thermal properties [1,2]. They have been widely used in electronics, medical equipment, aerospace, and other fields [3]. To meet the demand for producing complex-shaped copper parts with high dimensional accuracy, various metal manufacturing methods (pressure casting, investment casting) are used to create stable copper parts. These techniques have been stable for decades [4,5,6]. However, traditional metal fabrication techniques are limited to high-precision parts with complex internal structures. They are costly on account of the additional molds, long production cycles, and challenging post-processing, which limits the application of metal copper [7,8,9].

Three-dimensional printing, referred to as additive manufacturing (AM) technology, can realize complex structures. Compared to conventional manufacturing (metal casting, forging, and welding), AM has a considerable potential to manufacture objects with unique internal structures without being bound by specific processing rules [10,11,12]. In the last decade, many AM techniques have been reported for manufacturing complex metal parts with high dimensional accuracies, such as powder bed fusion (PBF) and selective laser melting (SLM) [13,14].

Yan et al. [15] used SLM to prepare stainless steel lattice structures with different volume fractions and designed a self-supporting structure to improve production efficiency. The lattice structure they produced was in good agreement with the original CAD model, but at the same time, they also obtained rough surfaces and large sizes. Tan et al. [16] prepared a TiNi lattice structure using LPBF. Based on the lattice’s geometric integrity and the microstructure evolution behavior, they optimized the pore and pillar sizes to reduce the deviation to less than two percent. The main reason for the microstructure deviation is the structural deformation caused by the heat input of LPBF technology. However, metallic copper’s inherent high thermal conductivity and high reflectivity to the laser make it challenging to fabricate high-precision parts at low cost. The digital light processing (DLP) AM process, which uses an ultraviolet-curable (UV) photographic slurry for manufacturing without regard to the heat conduction properties of the metal material, makes it possible to efficiently manufacture pure copper parts with high precision [17].

DLP is a relatively mainstream AM technology known for printing ceramic objects [18]^,^ with the advantages of high molding accuracy and short print cycles. DLP uses ultraviolet light of a specific wavelength to solidify the mixed slurry of photosensitive resin and metal powder [19]. The photopolymerizable slurry for this technology consists of a photosensitive resin containing monomers or oligomers, photoinitiators, dispersants, and powder materials. Currently, the technology is still under development for manufacturing metallic copper parts. Lee et al. [20] investigated the effect of photosensitive slurry containing metallic copper powder and the process on the electrical conductivity of the parts. Helical gears with a 200–300 nΩ·m resistivity were prepared under printing conditions with a layer thickness of 50 μm. Later, a slurry with 54 vol% copper powder content was also prepared by Sano et al. [21]. Copper photonic crystals with a layer thickness of 10μm were printed by micro-stereolithography. Roumanie et al. [17] printed pure copper parts with a digital light processing technique, focusing on the effect of different debinding and sintering atmospheres on the carbon and oxygen content of the parts. While problems related to the formation of metallic copper by stereolithography have been preliminarily solved, there are still many difficulties in realizing the commercialization of photocurable 3D printing metal materials. Few researchers have investigated the differences in dimensional accuracy caused by reduction photopolymerization during metal photocuring and the process parameters that enable the manufacture of the most petite sizes. In contrast to ceramic 3D printing, the high absorption of UV light by metallic copper implicitly affects the ability of UV light to penetrate the slurry and thus the dimensional accuracy, in addition to the light scattering phenomenon caused by the refractive index difference between the powder and the resin [22,23].

The metal material evaluated in this paper is pure copper, whose extremely high UV absorbance makes it more challenging to achieve photocuring than other metals. This study aims to establish the optimal printing parameters for the minimum size of metallic copper parts printed with DLP. It is worth noting that parameters such as exposure intensity, exposure time, layer thickness, and sweeper moving speed are vital characteristics for the widespread implementation of this technology. Therefore, the influence of these parameters on the dimensional accuracy of the printing model is systematically studied, and the dimensional deviation between the printing model and the design model is compared under different printing conditions. ANOVA was used to study the influence of various parameters and their synergistic effects on dimensional accuracy. After obtaining the most influential factors, the minimum feature size printing pure copper can achieve with this process was explored. The results of these studies could enable the preparation of complex copper structures with high dimensional accuracy by light-curing techniques. In addition, this study expands the application of dark-colored metal materials in stereolithography 3D printing and provides ideas for realizing high-precision 3D printing of metal materials.

## 2. Material and Methods

### 2.1. Raw Materials and Fabrication of Copper Samples

A metallic copper suspension and a commercial top-down DLP printer (Wavelength: 385 nm; Intensity: 230 mw/cm^2^) were both provided by Jiaxing CeramPlus Tech. Co. Ltd. (Jiaxing, China).

**Figure 1:**
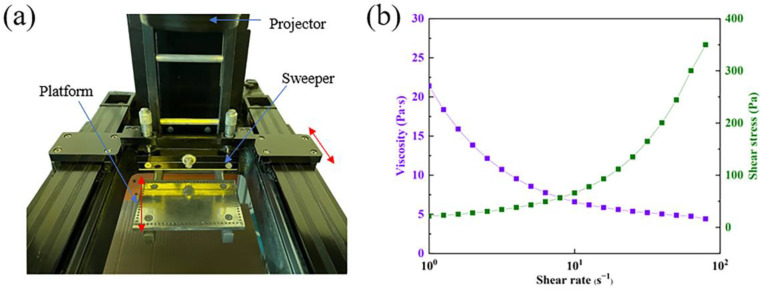
(**a**) Schematic diagram of top-down DLP printing and (**b**) rheological properties of copper slurry.

It is worth noting that the sweeper in Figure 1a is one of the unique working devices of the top-down printer. Its role is to precisely re-scrape a layer of liquid slurry on the top of the freshly solidified printed object. In this process, the slurry impacts the freshly printed green bodies, affecting the edge dimensions. This force is mainly determined by the viscosity of the slurry, which was sensitive to the sweeper moving speed, as seen in Figure 1b. Therefore, the sweeper moving speed will also be used as a printing parameter to investigate the effect on dimensional accuracy.

**Figure 2:**
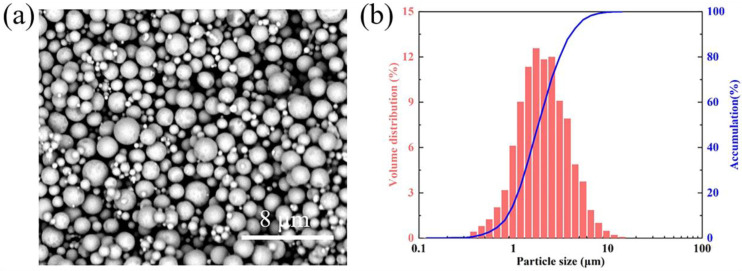
(**a**) The SEM image of copper powder and (**b**) the particle size distribution curve.

Figure 2 shows the electron microscopic image and particle size test of copper powder. The metal slurry with a solid content of 50 vol% is prepared from spherical copper powder. The copper powder consists of the primary particle size of D50 = 1.84 μm. The reason for choosing these two parameters is to ensure the density of the sample after printing. For photocuring printing technology, high solid content and finer powder are helpful to improve the various physical properties of the final product [24,25]. Copper powder’s particle size distribution and morphology were measured by a laser particle sizer (Winner 2000, Jinan Micro-Nano Particle Instrument Co., Ltd., Jinan, China) and a scanning electronic microscope (Phenom Pro, Phenom-World, Eindhoven, The Netherlands).

### 2.2. Print Pattern Design and Parameter Election

**Figure 3:**
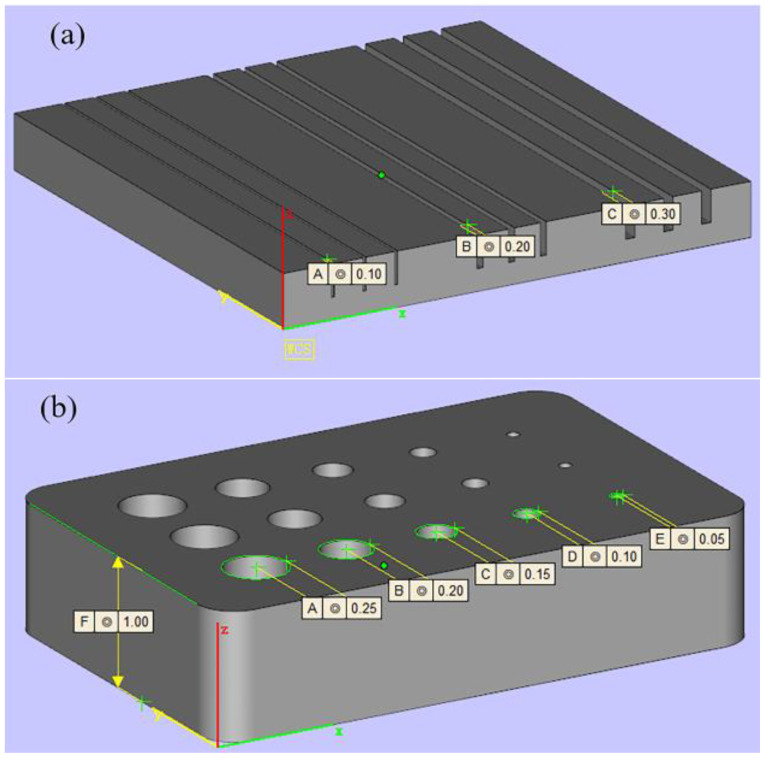
Design of models with different dimensions, (**a**) the width of the gap (d = 0.1 mm, 0.2 mm, 0.3 mm) and (**b**) the diameter of the hole (Ф = 0.1–0.5 mm).

To directly display the print size of metal slurry with different printing parameters, two models of gap width and hole diameter were designed for testing. The gap width was chosen in the range of 0.1–0.3 mm with a height of 2 mm, as shown in Figure 3a. The significance of this model is to explore the printing accuracy in the direction of the *x*-axis coordinate, which is suitable for printing lattice structures. The diameter of the hole model was selected in the range of 0.1–0.5 mm, and the thickness is 1 mm, as shown in Figure 3b. The diameter of the holes was investigated to test the degree of light scattering in both the *x*-axis and *y*-axis coordinates. The model reflected the optimal printing accuracy of the DLP process.

Chuchu Qian et al. [26] simulated the light scattering behavior caused by ceramic particles in stereo lithography manufacturing. They found that the wider the refractive index difference between the resin and the sandwich material, the more serious the light scattering manifested as increased in curing width. Michelle L et al. [27] tried to sum up the scattering equation of ultraviolet radiation in high-concentration suspension. Their research showed that ultraviolet radiation emitted into high-concentration suspension would produce both light scattering and absorption phenomena, and the square of the refractive index difference between particles and medium controlled the curing depth. It should be emphasized that their research object is ceramics powder with low UV absorbance, while it has been proved that the absorption of pure copper to UV light at 385 nm wavelength is more than 90% [28]. Photocurable printing is a process in which a photosensitive resin absorbs UV light and causes curing. The presence of copper powder competes with the resin for exposure energy.

Therefore, compared with the exposure parameters [29] of photocurable printing zirconia ceramics, higher exposure powder and longer exposure time were determined in Table 1 after the early paste test to ensure smooth printing. Nevertheless, at the same time, the higher exposure energy also means that the scattering of light caused by the difference in refractive index will be more severe.

This experiment selected exposure time, exposure intensity, layer thickness, and sweeper moving speed as the printing parameters to be explored. To improve the efficiency of the experiment, the range of parameters has been limited to a specific range by preliminary tests on the light curing depth of the green bodies. Exposure intensity refers to the power the light machine projects per unit of time. The synergistic action of the two parameters determined the total exposure energy received by the slurry. The thickness of the printing layer refers to the thickness of each layer of the green bodies designed during printing. A larger layer thickness means that a higher exposure energy is required. In addition, the role of the sweeper in the actual printing process is to apply the slurry evenly to the surface of the green bodies. The effect of the sweeper moving speed on the blank’s quality needs to be investigated.

### 2.3. Experimental Design and Characterization

In order to visualize the difference between the printed results and the designed model, deviations and RMSD were introduced into the experimental analysis, as shown in Equations (1) and (2). In this experiment, Minitab 21 was used to perform a multifactorial ANOVA and response table for means of RMSD. The multifactor ANOVA can predict the parameters that significantly affecting the printing accuracy and their synergistic effects by the deviations derived under different conditions.

(1) $$dev=x-x_{model}$$

(2) $$\mathrm{RMSD}=\sqrt{\frac{\sum_{i=1}^{n}{\left(x_{i}-x_{model}\right)}^{2}}{n}}$$

where dev is dimensional deviations, x is the average of the dimensions obtained and $x_{model}$ is the dimensions of models. RMSD can be used to compare the difference between the two data sets of the print model and the actual observed value. The smaller value indicates that the print result is closer to the design size, where n is the number of tests for each print size, and $x_{i}$ is the actual observed value for each print size.

## 3. Results and Discussions

### 3.1. Effect of Printing Parameters on Dimensional Accuracy

**Figure 4:**
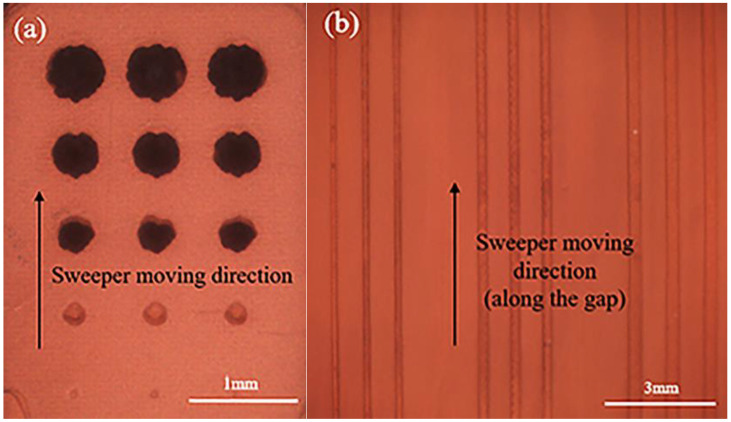
Gap samples (**a**) and hole samples (**b**) under the same printing conditions.

Figure 4 shows two models that were successfully printed using a commercial DLP printer under the same parameters. When comparing the difference between holes and gaps, it can be found that only 0.3–0.5 mm holes were successfully prepared, while 0.1–0.3 mm gaps existed. 

It is clear that the printing accuracy of both holes and gaps is affected by light scattering, but the shape of the printed holes and the diameter size indicate that the holes have more difficulty printing. This difference is mainly determined by the operating principle of the digital micro-mirror device (DMD) [30]. 

In imaging systems composed of DMD, the image needs to be imaged after rasterization. The aspect ratio of the DMD determines the aspect ratio of the reproduced image, and the image’s resolution is determined by the number of cells contained in the DMD [31]. For regular matrix images, the jaggedness of curved image edges due to the irregular filling of pixel points can be avoided by linear filling. Therefore, for the curved edges of the hole model, the irregular filling of pixel points is the main factor causing edge distortion. On the contrary, the gap model does not need to worry about the effect of jaggedness because the edges are regular and straight.

In addition, the sweeper moving direction is also an influencing factor for the different printing accuracy of the two models. The sweeper produces shear forces on the uncured slurry in the holes and gaps during operation. For the gap model in Figure 4b, the slurry in the gap will be carried out by the shear force because the gap position is parallel to the direction of the sweeper’s movement. On the contrary, the hole model has more light scattering effect due to the uncured slurry trapped in the hole, which causes more dimensional deviation. This also explains that the edge of the printed circular hole is more irregular than the printing gap.

**Figure 5:**
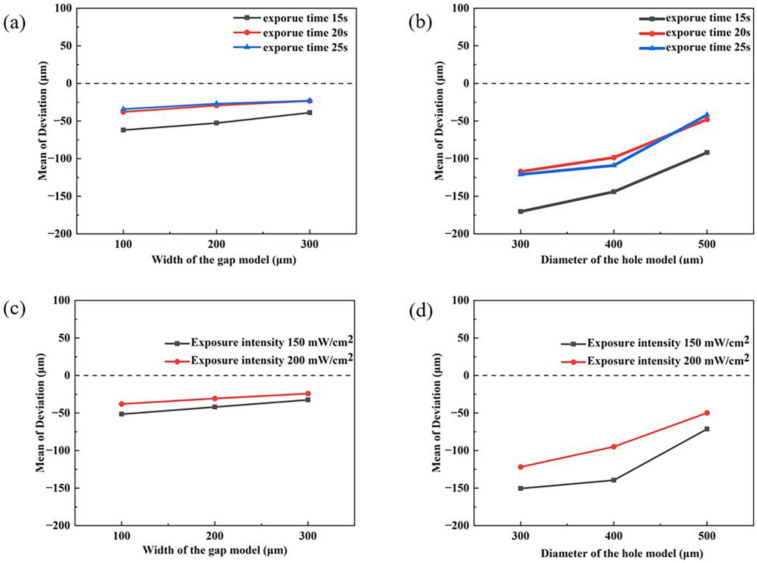
Dimensional deviation of gaps and holes for different printing parameters (**a**,**b**) the main effect plot for the exposure time, (**c**,**d**) the main effect plot for the exposure intensity.

Since 0.1 mm and 0.2 mm holes cannot be printed under some parameters, we only examine the response of the single parameter to dimensional deviation for 0.3–0.5 mm holes and 0.1–0.3 mm gaps. All the dimensional deviations are negative, as seen in Figure 5. The broadening of the exposed area causes this. The absolute deviations are significantly more significant for the hole model than the gap model. As the design size of the models becomes larger, the difference between them decreases. Furthermore, as the design size of the gap model increases from 0.1 mm to 0.3 mm, the dimensional deviation gradually decreases. It is even more evident in the aperture model. This can be explained as the increase in design size means that the unexposed area surrounded by the green bodies is more extensive, implicitly dispersing the additional exposure energy brought by light scattering. The edges of the exposed area are thus reduced in size due to the lack of energy. 

It can be observed in Figure 5 that the absolute dimensional deviation decreases accordingly during the increase of exposure intensity from 150 mW/cm^2^ to 200 mW/cm^2^. This indicates that the optimal exposure intensity exceeds 200 mW/cm^2^. In contrast, the increase in exposure time does not precisely follow this trend. For the gap model, the dimensional deviations were similar for different gap sizes at exposure times of 20 s and 25 s (Figure 5a). In addition, the dimensional variations for the hole model show that the absolute dimensional deviations are minor for 300 μm and 400 μm holes at an exposure time of 20 s. In addition, for the hole with a diameter of 500 μm, similar dimensional deviations were obtained at exposure times of 20 s and 25 s, respectively. The printing results of both models show that the most significant absolute dimensional deviation is at 15 s. For photopolymerizable copper slurry, shorter exposure times imply that fewer free radicals are generated by initiator decomposition. This further leads to an incomplete resin cross-linking reaction, resulting in insufficient strength of cured green bodies. During the sweeper’s movement, the green bodies’ surface will be damaged due to the shear force of the slurry. The damaged layer will remain retained at the edges, thus enlarging the dimensional deviation. 

In summary, the expected exposure time corresponding to a slight dimensional deviation can be printed between 20 s and 25 s without considering other factors. The total exposure energy, exposure energy, determined by both exposure time and intensity, largely determines the printing accuracy [32]. Therefore, an optimal exposure time and intensity must exist to optimize printing accuracy.

**Figure 6:**
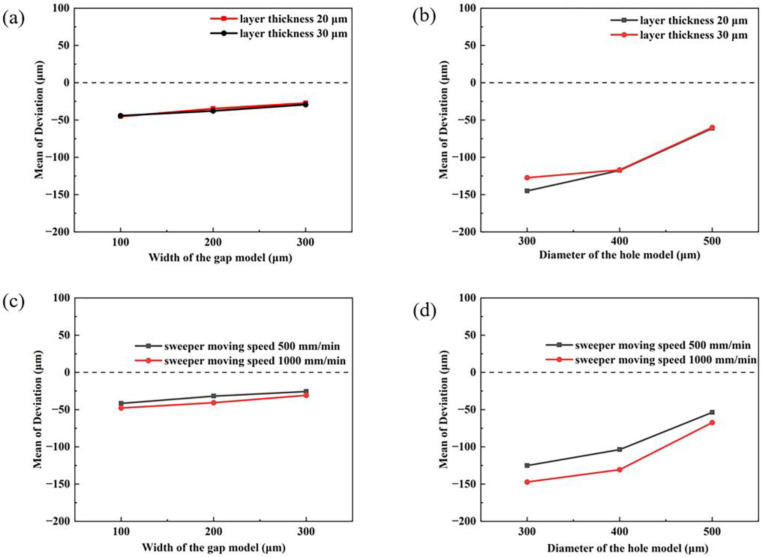
Dimensional deviation of gaps and holes for different printing parameters (**a**,**b**) the main effect plot for the layer thickness, (**c**,**d**) the main effect plot for the sweeper moving speed.

Figure 6 gives the dimensional deviations of the printed model at different layer thicknesses and sweeper moving speeds. Studying the DLP printing process further, it is clear that the actual curing depth of the green bodies is higher than the layer thickness of the model. This prerequisite ensures that the layers can bond tightly and maintain a particular strength. Beer–Lambert’s law determines the current relationship between the curing depth and exposure energy. Shen et al. [19] pointed out that this law can only predict the curing behavior within a specific range since the light transmission of the slurry continuously changes during the curing process. Figure 6 shows that the absolute dimensional deviation decreases as the layer thickness increases from 20 μm to 30 μm. This result is predictable, and the layer thickness of the model determines the degree of polymerization response in the z-direction for each raw layer. For a given exposure energy, a lower layer thickness means that polymerization in the z-direction is more likely to be saturated. In contrast, excess energy will lead to excessive polymerization effects in the x and y directions. Therefore, the choice of layer thickness must be determined in conjunction with the actual exposure energy and the curing depth.

In the actual production process, in addition to considering the photopolymerizable properties of the slurry itself, the sweeper as a mold that indirectly contacts the surface is also one of the parameters to be examined. The role of the sweeper is to bring the slurry uniformly to the surface of each layer of green bodies, so the moment of contact with the green bodies will generate an enormous shear force to cause damage to the sample. The damaged layer will move to the model’s edge and be affected by light scattering and secondary curing, resulting in negative dimensional deviations. To reduce the negative impact of shear force and to take into account the printing speed, a relatively slow sweeper moving rate of 500 mm/min is appropriate. It can be seen from Figure 6 that the absolute dimensional deviation of the two models is significantly smaller when the moving speed of the sweeper is 500 mm/min, which is caused by the shear stress of the slurry. This phenomenon can be explained by the slurry rheological characteristic curve in Figure 2b. With the increase of shear rate, the collision frequency between copper powder particles in the slurry and the resulting friction force increases correspondingly. Therefore, the slower moving speed of the sweeper will bring less shear force to the slurry to improve the printing accuracy.

### 3.2. ANOVA

To investigate the influence of printing parameters on dimensional accuracy, 24 different experiments were carried out. The values of input factors for each experiment are shown in Table 2.

The RMSD and mean response are shown in Table 2 and Table 3. The delta value can be used to compare the impact of different print parameters on the target indicator. A more considerable delta value indicates that the factor significantly influences the target index and may be essential. According to the results in Table 3, it is shown that ET, EI, and SMS have higher delta values, while layer thickness has the lowest delta values. This means layer thickness may be a minor factor compared to the other three parameters. In other words, changes in layer thickness have a limited effect on the dimensional accuracy of the printed model.

In order to verify the appropriateness of the above results, ANOVA was used to evaluate the factors affecting the dimensional deviation. Based on the 95% confidence level, factors with p-values less than or equal to 0.05 are considered critical effects. Therefore, the results of ANOVA in Table 4 show that exposure time, exposure intensity, and sweeper moving speed significantly affect on dimensional accuracy. The interaction between layer thickness, exposure time, and exposure intensity are significant process parameters, while there is no synergy between sweeper moving speed and other factors.

The results of ANOVA were similar to the response table for means of RMSD. When the layer thickness is considered a single factor, ANOVA results can be observed for different dimensional models with *p*-values greater than 0.05. This indicates its limited effect on dimensional accuracy [32]. This result is similar to Figure 6, where the dimensional deviation does not change with increasing layer thickness. On the contrary, their interaction significantly affects the deviation when it is linked to the exposure time and intensity. For ceramics, the increase in layer thickness reduces the effect of light scattering, as the photoinitiator absorbs more exposure energy in the z-direction [33], while for metallic copper slurry, the high absorbance of pure copper powder weakens the effect of this layer thickness. 

In the DLP process, exposure time, exposure intensity, and layer thickness will be considered overall parameters because they determine the average energy density per layer of green bodies in the z-direction. Therefore, the layer thickness is generally considered a constant value compared to the exposure time and intensity, which have a higher influence. The opposite is true for sweeper moving speed, as it significantly affects dimensional deviation when considered as a single factor. However, its interaction with the other factors has a *p*-value greater than 0.05. In general, sweeper moving speed as a mechanical parameter determines the effect of slurry shear on the green bodies. The shear force is rarely associated with the curing process parameters for the same photocurable slurry. Therefore the synergistic effect of the sweeper with other factors is not significant.

In summary, dimensional deviations are correlated with various factors in different ways. Therefore, an optimal set of parameters exists to obtain the best value of the dimensions. Considering the above single-factor influence results and the data from ANOVA, the exposure time and intensity should be limited to 20–25 s and 200–230 mW/cm^2^, respectively, to print holes with minor dimensional deviations and optimum dimensions. In addition, the layer thickness should be maintained at 30 μm and the sweeper moving speed at 500 mm/min. When the layer thickness changes, the exposure time and intensity, which are synergistic, will also change. A reduction in sweeper moving speed will increase the printing time with a slight deviation. Therefore, to ensure the experiments’ rationality and to increase the efficiency of the experiments, constant values were taken for both parameters.

### 3.3. Further Optimization

This step aims to further reduce light scattering effects and dimensional deviations to print optimal holes. Based on the conclusions of the initial optimization step, the exposure time and exposure intensity were further optimized while keeping the layer thickness and sweeper moving speed constant. An optimized design was used, and 24 runs were performed. Table 5 summarizes the experimental factors and the results obtained for each experiment. The experimental results show that, as the printing parameters were varied, holes of 200 μm were always successfully printed, and their absolute dimensional deviation was within 100 μm. For different diameters of holes, the minimum deviation was achieved with an exposure intensity of 220 mW/cm^2^ and an exposure time of 21 s.

**Figure 7:**
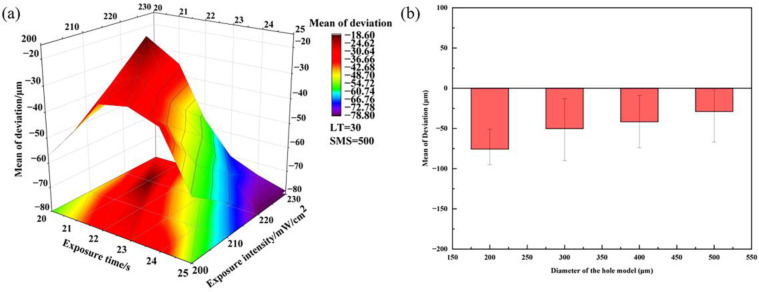
(**a**) Response surface plot of the mean deviation and (**b**) deviation of hole size.

As seen in Table 5, for the hole model with a design size of 200 μm, the smallest hole printed was 105 μm (ET = 25 s, EI = 220 mW/cm^2^). The printing accuracy of the 200 μm aperture has been dramatically improved compared to the initial screening experiments. This indicates that the optimization range of its exposure time, exposure intensity, layer thickness, and sweeper moving speed is reasonable. Figure 7 shows the response surface plot of the mean deviation and a graphical representation of the dimensional deviation for different hole diameters.

**Figure 8:**
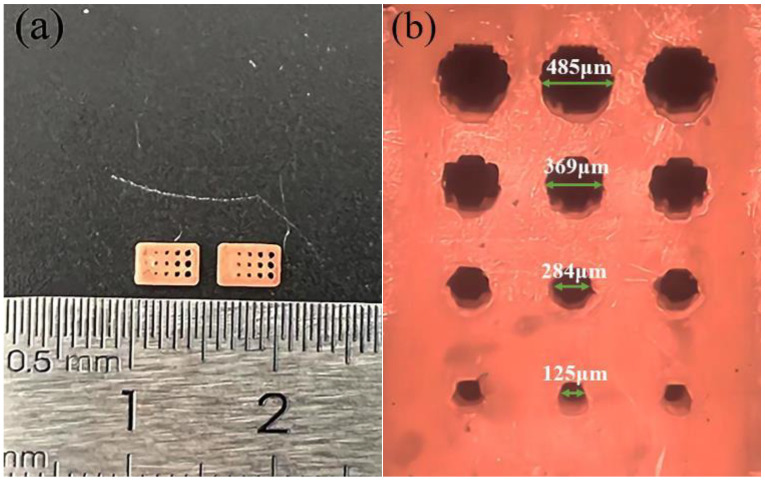
(**a**) Optimized hole image view and (**b**) SEM image.

As a graphical representation of a regression model, the response surface method can be used to precisely investigate the relationship between factors and response values by designing a reasonably small number of trials and quickly determining the optimal conditions for a multifactor system. Figure 7a shows that the initial increase in the mean size deviation is a sizeable maximum level and then decreases slowly with increasing exposure time. In addition, the higher level of mean dimensional deviation is maintained within a specific range under the conditions of varying ET and EI. This phenomenon can be explained by the fact that the total exposure energy, determined by ET and EI, is an essential factor affecting the green bodies’ dimensional deviation. When the total exposure energy is far from the optimal conditions, it reduces the average dimensional variation. The optimal printing parameters are 21 s exposure time and 220 mW/cm^2^ exposure intensity. This observation is consistent with the experimental results of group 7 in Table 5. All three designs of holes with a size of 200 μm have varying degrees of distortion (Figure 8), mainly in negative deviations. Extensive references indicate that the excessive curing width is mainly attributed to the difference in refractive index between resin and powder [34,35,36].

In the case of metallic copper, the copper powder has an extremely high absorbance of UV light at a wavelength of 385 nm (copper DLP). This means that when UV light penetrates the surface of the slurry, some of the UV light will be attracted to the copper powder at the model’s edge. This will lead to the migration of free radicals and polymerization reactions. Figure 7b shows the dimensional deviations of the pore size for different exposure parameters. As can be seen, the dimensional deviation for all samples is less than 100 μm. The 200 μm hole’s average dimensional deviation is significantly larger than the sizes of the other three holes.

Furthermore, the slightest deviation printed for the 200 μm hole is around 50 μm, and the average size deviation is about 75 μm (Figure 8b). The larger holes (Ф_Model_ = 300 μm, 400 μm, and 500 μm) have a minimum deviation of 20 μm or less, and the minimum deviation decreases as the diameter of the print hole increases. As the aperture size increases, the scattering area also increases. The scattering effect decreases as the spatial distribution of UV light scattered during the curing process increases.

In summary, the printing accuracy of metallic copper slurry is well improved after this optimization step. In addition, all 200 μm holes were successfully printed, and the optimum results obtained for 200 μm holes provide applicability for various high-precision metal part applications. A 100 μm minimum wall thicknesses for pure copper are currently reported by Qu et al. [37] The printing accuracy for holes has not yet been mentioned. In general, holes are more challenging to print than wall thicknesses control, which was only affected in one direction. In addition, Conti et al. [38] reported that the design size of the smallest hole that can be printed out by using DLP technology to prepare zirconia ceramics is 200 μm (LT = 25 μm), which is close to the size of the smallest hole that can be printed in this report.

### 3.4. Properties of Metal Copper

**Figure 9:**
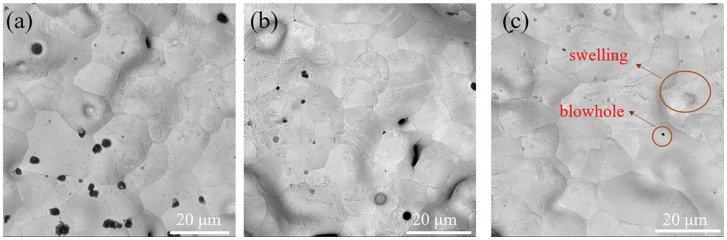
Surface microstructure of Cu sintered parts under different holding time: (**a**) 2 h, (**b**) 4 h and (**c**) 6 h.

Figure 9 shows the sintering density of pure copper held at 1050 °C for 2 h, 4 h, and 6 h, respectively. The results show apparent pores on the sintered parts with a holding time of 2 h, and the sintered parts with a holding time of 4 h and 6 h are denser. The appearance of stomata is related to the stomatal channels that remain after the volatilization of organic matter in the green bodies. With the increase in temperature, part of the remaining pore channels was not filled due to the growth of grains, resulting in the bulging phenomenon, and the other part was left on the sample surface to become pores due to insufficient holding time.

**Figure 10:**
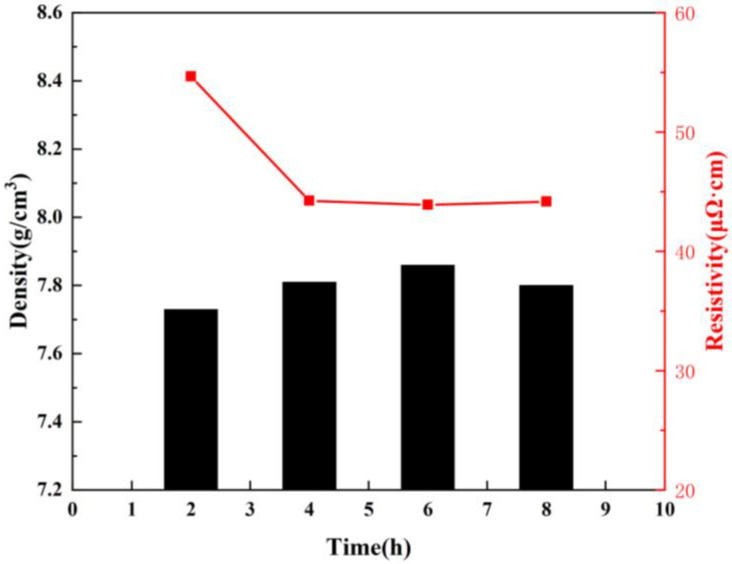
Density and resistivity of Cu sintered parts under different holding time.

Figure 10 shows the resistivity of the three sintered parts. Corresponding to the SEM image, the lowest resistivity of the sintered part with a holding time of 6 h is 43.89 μΩ·cm. In general, the three groups of samples were not sufficiently densified. This is related to the choice of the atmosphere when the green bodies are discharged. In order to ensure that the copper is not oxidized during the heating process, a protective atmosphere is selected, which also allows the residual carbon to remain in the sample without reaction conditions. Although this is lower than the reported density [17], the sintering method did not select an oxidation route, which allowed the printing accuracy of the copper to be retained in the sintered finished product, which was not previously reported.

## 4. Conclusions

This study aims to realize high-precision photocurable 3D printing of pure copper materials and improve the printing accuracy while determining the significant influencing factors by combining ANOVA with RMSD. The results showed that a gap of 100 μm and a hole of 200 μm diameter were successfully printed, and all dimensions of the printed model were more extensive than the design value of the model. This is caused by the high absorbance of metallic copper to 385 nm UV light and the refractive index between the powder and resin. A multifactor ANOVA was used to predict the degree of influence of different parameters on dimensional deviations. Exposure time and intensity are considered the root cause of significant size deviation. Their synergistic effect with layer thickness determines the total UV irradiation energy received by each layer of green bodies. The thickness itself is not a significant factor in printing accuracy. The print quality can be affected only when it is associated with exposure time and exposure intensity. After optimizing the parameters (exposure time = 21 s, exposure intensity = 220 mW/cm^2^, layer thickness = 30 μm, sweeper moving speed = 500 mm/min), a hole model with a height of 1mm and a design diameter of 200 μm is successfully printed. The minimum size deviation is 51 μm, and the average size deviation is 75 μm. This result shows that even pure copper powder with high absorbance can be used directly for reduction photopolymerization printing and maintain excellent printing accuracy.

It should be emphasized that this paper mainly studies the printing process of DLP- forming technology in detail, and another essential link of this technology is the heat treatment of the green bodies sintering. The shrinkage caused by green bodies sintering will undoubtedly result in a smaller print size. For the photocurable copper paste used in this paper, the shrinkage is about 10~15 wt%, and the size of the corresponding pore model will shrink to about 100 μm. This is a process worth watching. Future work could explore the effect of parameters related to heat treatment on the dimensional accuracy of green bodies after sintering.

## Figures and Tables

**Table 1:** Range of printing parameters.

Exposure Time (ET) (s)	Exposure Intensity (EI) (mW/cm^2^)	Layer Thickness (LT) (μm)	Sweeper Moving Speed (SMS) (mm/min)
15, 20, 25	150, 200	20, 30	500, 1000

**Table 2:** RMSD for the dimensional accuracy of the model with an aperture of 300 μm.

No.	ET (s)	EI (mW/cm^2^)	LT (μm)	SMS (mm/min)	RMSD (μm)
1	15	150	30	500	213.06
2	15	150	30	1000	228.02
3	15	150	20	500	144.12
4	15	150	20	1000	162.21
5	15	200	30	500	167.06
6	15	200	30	1000	192.10
7	15	200	20	500	111.19
8	15	200	20	1000	146.15
9	20	150	30	500	145.21
10	20	150	30	1000	196.04
11	20	150	20	500	130.01
12	20	150	20	1000	148.03
13	20	200	30	500	40.22
14	20	200	30	1000	53.04
15	20	200	20	500	102.02
16	20	200	20	1000	123.03
17	25	150	30	500	88.22
18	25	150	30	1000	94.17
19	25	150	20	500	118.01
20	25	150	20	1000	140.02
21	25	200	30	500	42.10
22	25	200	30	1000	70.03
23	25	200	20	500	202.02
24	25	200	20	1000	214.03

**Table 3:** Response table for means of RMSD.

Level	ET	EI	LT	SMS
1	170.5	150.6	145.1	125.3
2	117.2	121.9	127.4	147.2
3	121.1			
Delta	53.3	28.7	17.6	22.0
Rank	1	2	4	3

**Table 4:** ANOVA for the size deviation of the model.

Source	*p*-Values for Gap Model			*p*-Values for Hole Model		
	d_Model_ = 100 μm	d_Model_ = 200 μm	d_Model_ = 300 μm	Ф_Model_ = 300 μm	Ф_Model_ = 400 μm	Ф_Model_ = 500 μm
ET	0.000	0.000	0.000	0.001	0.020	0.000
EI	0.000	0.000	0.003	0.006	0.003	0.002
LT	0.623	0.117	0.365	0.054	0.953	0.806
SMS	0.017	0.001	0.036	0.022	0.037	0.022
ET & EI	0.002	0.000	0.028	0.003	0.232	0.010
ET & LT	0.030	0.352	0.300	0.000	0.039	0.040
ET & SMS	0.120	0.984	0.980	0.900	0.823	0.467
EI & LT	0.004	0.005	0.005	0.001	0.009	0.015
EI & SMS	0.523	0.158	0.877	0.968	0.815	0.832
LT & SMS	0.733	0.210	0.447	0.903	0.907	0.832

**Table 5:** Design and experimental results of the improved printing experiment for hole model.

	ET	EI	Ф = 200 μm	Ф = 300 μm	Ф = 400 μm	Ф = 500 μm
1	20	200	113	248	353	457
2	20	210	125	253	362	464
3	20	220	127	259	367	472
4	20	230	137	265	384	489
5	21	200	132	261	373	453
6	21	210	141	270	380	479
7	21	220	149	287	391	498
8	21	230	136	279	353	492
9	22	200	138	275	375	485
10	22	210	145	279	380	487
11	22	220	132	253	359	479
12	22	230	113	233	350	478
13	23	200	140	270	385	485
14	23	210	124	266	355	483
15	23	220	129	228	353	473
16	23	230	111	219	338	452
17	24	200	125	284	369	485
18	24	210	119	241	344	475
19	24	220	108	226	341	457
20	24	230	107	210	335	448
21	25	200	112	236	348	482
22	25	210	109	220	347	460
23	25	220	105	216	326	438
24	25	230	106	218	329	433

## Data Availability

The data underlying this article will be shared on reasonable request from the corresponding author.
